# Investigating the deregulation of metabolic tasks via Minimum Network Enrichment Analysis (MiNEA) as applied to nonalcoholic fatty liver disease using mouse and human omics data

**DOI:** 10.1371/journal.pcbi.1006760

**Published:** 2019-04-19

**Authors:** Vikash Pandey, Vassily Hatzimanikatis

**Affiliations:** Laboratory of Computational Systems Biotechnology, EPFL, Lausanne, Switzerland; US Army Medical Research and Materiel Command, UNITED STATES

## Abstract

Nonalcoholic fatty liver disease (NAFLD) is associated with metabolic syndromes spanning a wide spectrum of diseases, from simple steatosis to the more complex nonalcoholic steatohepatitis. To identify the deregulation that occurs in metabolic processes at the molecular level that give rise to these various NAFLD phenotypes, algorithms such as pathway enrichment analysis (PEA) can be used. These analyses require the use of predefined pathway maps, which are composed of reactions describing metabolic processes/subsystems. Unfortunately, the annotation of the metabolic subsystems can differ depending on the pathway database used, making these approaches subject to biases associated with different pathway annotations, and these methods cannot capture the balancing of cofactors and byproducts through the complex nature and interactions of genome-scale metabolic networks (GEMs). Here, we introduce a framework entitled **M**inimum **N**etwork **E**nrichment **A**nalysis (MiNEA) that is applied to GEMs to generate all possible alternative minimal networks (MiNs), which are possible and feasible networks composed of all the reactions pertaining to various metabolic subsystems that can synthesize a target metabolite. We applied MiNEA to investigate deregulated MiNs and to identify key regulators in different NAFLD phenotypes, such as a fatty liver and liver inflammation, in both humans and mice by integrating condition-specific transcriptomics data from liver samples. We identified key deregulations in the synthesis of cholesteryl esters, cholesterol, and hexadecanoate in both humans and mice, and we found that key regulators of the hydrogen peroxide synthesis network were regulated differently in humans and mice. We further identified which MiNs demonstrate the general and specific characteristics of the different NAFLD phenotypes. MiNEA is applicable to any GEM and to any desired target metabolite, making MiNEA flexible enough to study condition-specific metabolism for any given disease or organism.

## Author summary

This work aims to introduce a network-based enrichment analysis using metabolic networks and transcriptomics data. Previous pathways/subsystems enrichment methods use predefined gene annotations of metabolic processes and gene annotations can differ based on different resources and can produce bias in pathway definitions. Thus, we introduce a framework, Minimum Network Enrichment Analysis (MiNEA), which first finds all possible minimal-size networks for a given metabolic process/task and then identifies deregulated minimal networks using deregulated genes between two conditions. MiNEA also identifies the deregulation in key reactions that are overlapped across all possible minimal-size networks. We applied MiNEA to identify deregulated metabolic tasks and their synthesis networks in the steatosis and the nonalcoholic steatohepatitis (NASH) diseases using a metabolic network and transcriptomics data of mouse and human liver samples. We identified key regulators of NASH for the synthesis networks of hydrogen peroxide and ceramide in both humans and mice. We also identified opposite deregulation in NASH for the phosphatidylserine synthesis network between humans and mice. MiNEA finds synthesis networks for a given target metabolite and due to this it is flexible to study deregulation in different phenotypes. MiNEA can be widely applicable for studying context-specific metabolism for any organism because the metabolic networks and context-specific gene expression data are now available for many organisms.

## Introduction

Nonalcoholic fatty liver disease (NAFLD) is the most common cause of liver disease in western countries [1], affecting an estimated 25% to 45% of the general US population, though this percentage is much higher among individuals suffering from obesity and diabetes [1, 2]. There are two categories of NAFLD: steatosis, which is an accumulation of fat in the liver, and nonalcoholic steatohepatitis (NASH), which consists of the additional presence of liver inflammation and hepatocellular injury with or without fibrosis [2].

Gene deregulation occurs when the cell no longer maintains precise control over certain genes, and therefore expressed proteins, and is associated with various diseases such as NAFLD. Determining which genes are deregulated in disease and how these genes are deregulated can increase the understanding of any disease as well as provide new pathways for therapeutic treatments. To study gene deregulation, pathway enrichment analysis (PEA) uses maps of metabolic processes and subsystem reactions to determine if a given list of genes (gene set) is associated with a certain biochemical pathway, shedding important conceptual insight into gene deregulation. Many PEA algorithms, such as ConsensusPathDB [3] and Piano [3, 4], have been developed to identify biological processes based on gene sets, while other algorithms, such as IMPaLA [5], are based on both gene and metabolite sets. While these algorithms can successfully provide insights about various disease phenotypes, they use predefined metabolic maps that are limited to our current knowledge of these hugely intricate pathways and can differ depending on the pathway database used, meaning they could miss information about the wide range of complex metabolic interactions. These approaches are also subject to biases associated with the different pathway annotations, and they cannot capture the balancing of cofactors and byproducts through the complex nature and interactions of genome-scale metabolic networks (GEMs).

To overcome this, a method has been developed based on elementary flux modes (EFMs), which are non-decomposable flux distributions in metabolic networks [6].These flux distributions indicate a conceptual description of metabolic pathways. This method computes EFMs from a given GEM and uses tissue-specific transcriptomics to identify a subset of tissue-specific EFMs. However, the enumeration of all EFMs in a GEM can be computationally intractable, and EFMs are not necessarily specific to a target metabolite synthesis, meaning that targeted pathways are not always able to elucidated using EFMs.

Modeling methods of biological networks, such as metabolic, signaling, and protein-protein interaction can be used as a framework to study biological functions of the liver for the prevention and treatment of liver-associated disease [7]. For example, one can use graph-based methods (GBM) that require network topologies and gene expression profiles as inputs to extract deregulated subnetworks that occur between two conditions [8–10]. These methods have been applied to protein-protein networks for prostate cancer study, and they are potentially applicable to metabolic networks. GBM methods use graph-theoretic properties on network topologies but miss information about additional constraints, such as mass balance [11] and thermodynamics [12]. Ideally, the results for these studies would include a set of mass balanced subnetworks that could be used to understand the carbon, energy, and redox flows from precursor metabolites to target metabolites and complex metabolic tasks.

Here, we propose a method called **Mi**nimal **N**etwork **E**nrichment **A**nalysis (MiNEA) that compares two conditions using transcriptomics, proteomics, and metabolomics to identify deregulated minimal networks (MiNs), which are reactions pertaining to various metabolic subsystems that can synthesize a target metabolite. The MiNEA algorithm works by formulating metabolic tasks (MTs) to mimic the various NAFLD phenotypes, such as lipid droplet formation, lipoapoptosis, liver inflammation, and oxidative stress. These MTs include the known set of metabolites necessary to form the modeled phenotype. The cell can use different pathways to form the same metabolites and create the same phenotypes, and the purpose of MiNEA is to identify deregulated alternative routes for a given MT between two conditions, such as a control and treatment. Additionally, MiNEA uses these MTs to compute alternative thermodynamically feasible MiNs applying thermodynamic constraints [12, 13] to a mouse GEM [14].

We used mouse and human liver sample expression data [1, 15] and identified deregulated metabolic processes in mice and humans that potentially lead to the NAFLD phenotypes. MiNEA identified an upregulation in the oxidative stress synthesis network in mice, but this network was found to be downregulated in humans. The cholesterol and triacylglycerol synthesis networks were deregulated in humans only, while the ceramide synthesis network was only deregulated in mice. We found downregulated reactions in the synthesis network for cholesteryl esters, cholesterol, and alanine in both humans and mice. We further identified downregulated reactions in the superoxide anion (SOA) synthesis network in humans, specifically in NASH as opposed to steatosis, while upregulation was found in the SOA synthesis network in mice. This perturbation through the SOA synthesis network in NASH suggested an unbalanced ceramide synthesis, and studies have shown that the ceramide is a key regulator of apoptosis and promotes fibrosis in the hepatic steatosis model [16, 17].

MiNEA can generate MiNs for any target metabolite, e.g. a metabolite produced under specific phenotypes or a biomass building block [18] that is needed for cell growth. Additionally, MiNEA can integrate condition- and context-specific omics data to understand the deregulated phenotypes associated with a set of differentially expressed genes. These characteristics make MiNEA a versatile tool for exploring and understanding different metabolic phenotypes.

## Results and discussions

### Experimental details for MiNEA

Nonalcoholic fatty liver disease (NAFLD) has been defined as a metabolic disease associated with insulin-resistance syndrome [19]. To study the differences seen in NAFLD phenotypes between mouse and human manifestations, we collected human expression data from the three diagnosis groups, normal (N), steatosis (S), and nonalcoholic steatohepatitis (NS), and mouse expression data from control and DDC-supplemented diet conditions for three genetically different mouse strains, AJ, B6, and PWD. DDC-supplemented diet reproduces steatosis and NASH phenotypes (see Materials and Methods for detail). We refer to these data as *human expression data* and *mouse expression data* throughout this section and integrate it into a mouse model iMM1415 [14] that is constructed based on a human GEM Recon1 [20]. Sigurdsson and colleagues found that the mammalian organism with the highest number of genes homologous to Recon 1 genes was the mouse (*Mus musculus*) (1,415 genes, 97%). We compared iMM1415 and Recon1 and found that the iMM1415 shares 98% of reactions with Recon1 and in the remaining 2% reactions more than 75% (more than 1.5% of total reactions) were associated with the extracellular transport mechanism. This suggests that Recon1 and iMM1415 have a very similar metabolism. Computed minimal networks do not change between Recon1 and IMM1415 due to less variability between the two models. Thus, we use iMM1415 as a generic model. If we impose tissue specific constraints or organism specific external medium, we expect some changes in the minimal networks, mainly in the number of alternative networks. However, such constraints involve a lot of uncertainty and we chose to use a generic model and our results here can be viewed as an *upper bound* on the possible solutions. We analyzed both human and mouse MTs using the generic model (iMM1415) with the assumption that both types of mammalian cells have a similar metabolism (See S1 Text). In the following sections, we analyzed the deregulation of minimal networks in core reactions and enriched minimal networks based on the deregulation (up- or downregulated) genes.

### Minimal networks

GEMs represent an entire cellular metabolic network in the form of mathematical constraints [21]. These network reconstructions have grown rapidly in the last decades, and now many GEMs for different organisms are available [22]. GEMs and condition-specific experimental data can be employed to generate and test hypotheses using the Minimal Network Enrichment Analysis (MiNEA) framework that has been developed in this study. Compared to a method that optimizes only some part of GEMs to find subnetworks [23], MiNEA optimizes whole GEMs to find minimal networks.

To examine the data that can be derived from MiNEA in terms of NAFLD, we first wanted to compute MiNs for given MTs (see Materials and Methods) that were significant phenotypes in NAFLD. Instead of representing a main linear route between precursors and targets of MTs, a MiN is composed of many subsystems integrated into subnetworks that must be active, meaning that reactions of subnetworks carry flux, to fulfill the target MT. The MiNEA algorithm facilitates enumeration of alternative MiNs and provides more flexibility to the analysis of different metabolic phenotypes and their respective environmental and genetic perturbations. The use of MTs for analysis with MiNEA allows this method to be more easily generalized and applied to the study of other metabolic phenotypes and diseases.

A summary of the MiNs calculated for MTs (see Materials and Methods) are shown in Table 1. The shortest MiNs were for the synthesis of hydrogen peroxide (H_2_O_2_) (network size = 37), and the longest were for the synthesis of cholesteryl ester (network size = 131; Table 1). We found the greatest number of possible alternatives for the hexadecanoate (HDCA) synthesis and the least number possible for the phosphatidylserine (PS) synthesis (Table 1), suggesting that the HDCA synthesis is more flexible and the PS synthesis less flexible towards alternative formation compared to the rest of the MTs. Reactions that overlapped between all alternative MiNs were called high-frequency reactions (HFRs), and the percentage of HFRs shows how similar or divergent a MiN is compared to the other MiNs. The percentage of HFRs was between 62% to 91% across the MTs (Table 1). In the superoxide anion (SOA) and the hydrogen peroxide (H_2_O_2_) synthesis, 62% and 89% of the reactions were HFRs, respectively, which suggests that the alternative MiNs for the SOA synthesis were more divergent compared to the H_2_O_2_ synthesis.

**Table 1:** Summary of MiNs found for the target metabolites comprising the various NAFLD phenotypes. Synthesis of a target metabolite represents a MT. For each MT, we enumerated alternative MiNs. The number of reactions in each MiN represents the size of the MiN. “# Alt” and “# MT HFRs” represent the number of alternatives and the number of overlapping reactions between all alternatives of a given MT, respectively. The symbol “#Phenotypic HFRs” represents the number of overlapping reactions from all alternatives of a set of MTs that are associated with a given phenotype.

Phenotypes	Metabolic	Short	Min Size	#	# MT	%MT	# Phenotypic
	Tasks (MTs)	Name	# rxns	Alt	HFRs	HFRs	HFRs
Oxidative	Hydrogen peroxide	H_2_O_2_	37	126	33	89	16
Stress	Superoxide anion	SOA	42	144	26	62	
Apoptosis and	Alanine	ALA	98	39	84	86	36
Inflammation	Ceramide	CRM	115	8	105	91	
	Glutamine	GLU	116	34	83	72	
Lipid droplet	Cholesterol	CHOL	121	60	108	89	27
	Cholesteryl ester	CHOL_ES	131	237	112	85	
	Diacylglycerol	DAG	82	232	67	82	
	Hexadecanoate	HDCA	75	266	61	81	
	Phosphatidic acid	PA	83	245	65	78	
	Phosphatidylinositol	PAIL	94	237	68	72	
	Phosphatidylethanolamine	PE	128	7	103	80	
	Phosphatidylserine	PS	125	6	98	78	

**Fig 1:**
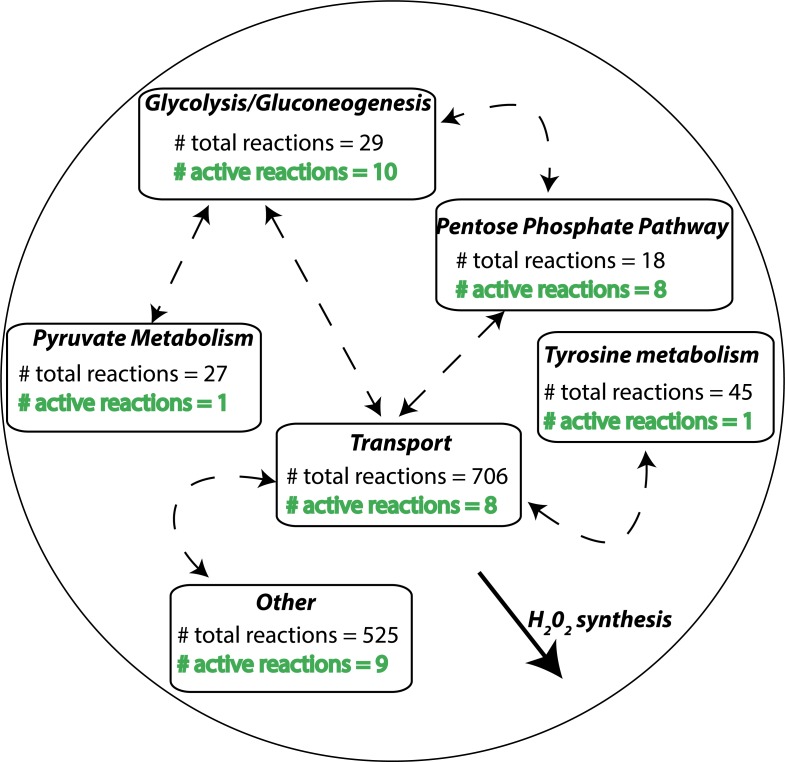
A minimal network (MiN) and high-frequency reactions (HFRs). A MiN of H_2_O_2_ synthesis is illustrated. Each box represents a different metabolic subsystem/pathway. The number of total reactions listed in each box represents the annotated reactions for that subsystem. Active reactions carry non-zero flux, and the number of these present in each subsystem is shown in green text.

As an expanded example, a MiN from the H_2_O_2_ synthesis comprises many reactions from various metabolic subsystems (Fig 1). Each MiN represents a group of active reactions within multiple metabolic subsystems/pathways that are required for a MT, while a pathway is a group of annotated reactions. In the example in Fig 1, most of the active reactions for the H_2_O_2_ synthesis are from the pentose phosphate pathway (PPP) and the glycolysis/gluconeogenesis pathway. PEA differs in that it identifies a marked deregulation in a pathway as compared to MiNEA, which identifies deregulation in a MiN that could include multiple pathways.

**Fig 2:**
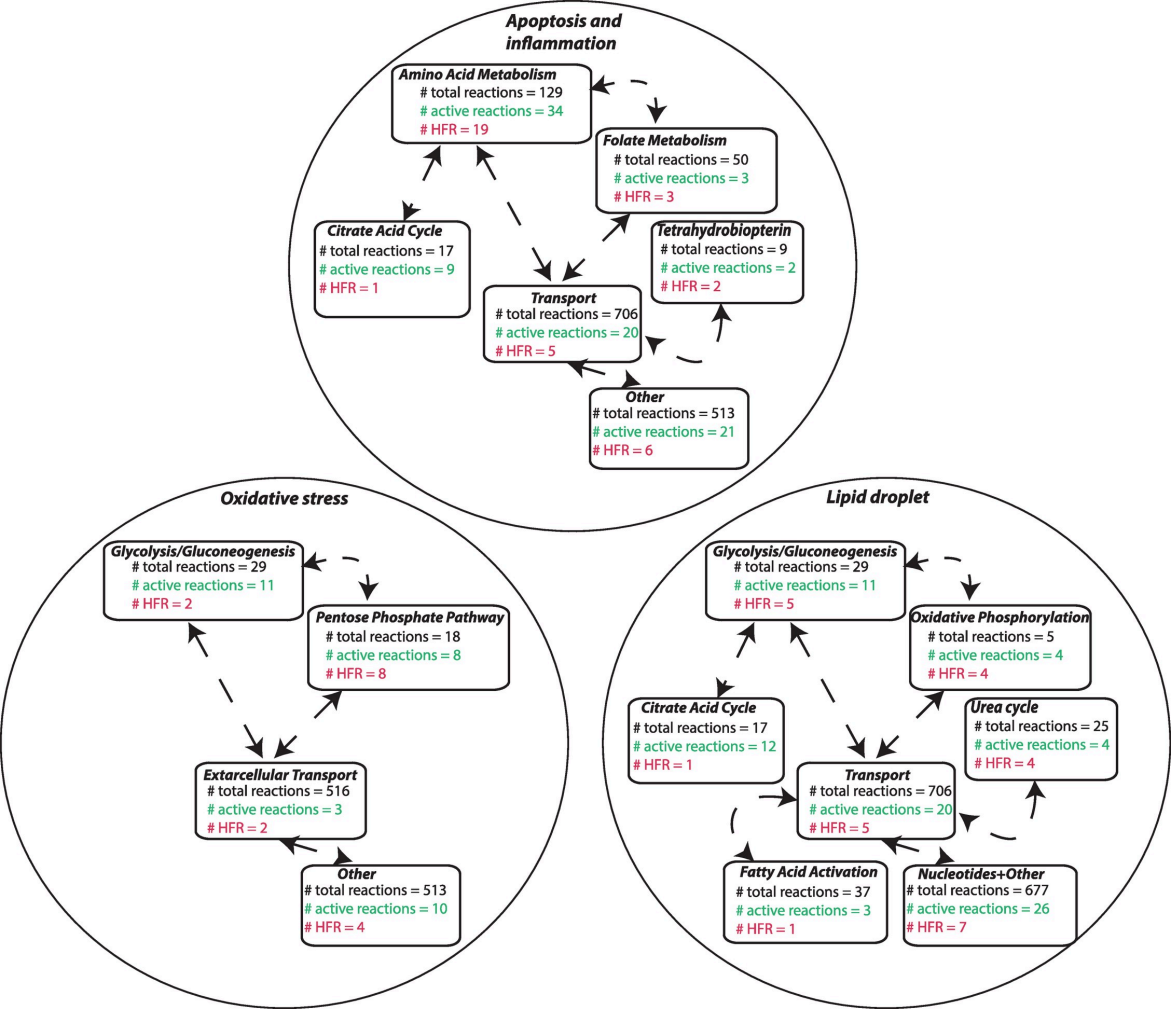
HFRs for all the phenotypes. The number of HFRs in the various pathways is shown for the oxidative stress, apoptosis and inflammation, and lipid droplets phenotypes. See Fig 1 caption for a detailed explanation of the chart.

We further analyzed the HFRs within each phenotype and found that the most represented metabolic pathway in the apoptosis and inflammation (AI) phenotype was related to amino acid metabolism (# phenotypic HFRs = 36; Table 1; Fig 2). Interestingly, while the lipid droplet (LD) phenotype has many associated MTs, it shares 27 reactions in which 5 reactions are from the glycolysis/gluconeogenesis pathway (Fig 2). These shared reactions can be constitutive candidates for the LD phenotypes. The HFRs forming the subsystems in Fig 2 are potential candidates for the key regulators of the NAFLD phenotypes.

### Deregulation in HFRs

The HFRs within each MT create a topology of reaction hubs in metabolic networks that can be similar to protein hubs in protein-protein interaction networks [24, 25]. The misregulation of these hubs in disease can explain the origin of disease phenotypes. Therefore, we performed an analysis of HFR deregulation to study the NAFLD-specific phenotypes. We identified the up- and downregulated reactions from mouse and human expression data (described in S1 Text) and analyzed the HFRs for which the associated genes were found to be deregulated. In humans, the number of downregulated HFRs was higher compared to upregulated HFRs for the NS *vs* N and NS *vs* S across all phenotypes (Table 2). In mice, however, the NS *vs* N, NS *vs* S, and S *vs* N comparisons showed a higher number of upregulated HFRs compared to downregulated ones for the oxidative stress (OS) phenotype, but for the LD and AI phenotypes, we observed similar patterns of up- and downregulated HFRs to humans (Table 2).

**Table 2:** The deregulated HFRs across all comparisons. Numbers in the table indicate the number of up- and downregulated HFRs. In mice, N represents the control diet (N≈AJ, N≈PWD, or N≈B6 strain fed the control diet). Under the DDC-supplemented diet, AJ mice tended towards NASH phenotypes and PWD mice tended towards steatosis phenotypes. Thus, in mice, the symbols NS stand for AJ and S stand for the PWD, all with DDC-supplemented diet (NS≈AJ with DDC-supplemented diet; S≈PWD DDC-supplemented diet).

			M	O	U	S	E		
			N		NS		S		
			up	down	up	down	up	down	
	** **	**OS**			7		2		** **
H	**N**	**AI**				9	3	5	**N**
U	** **	**LD**			2	3	1	2	** **
M	** **	**OS**		5			3	1	** **
A	**NS**	**AI**	3	5			6	10	**NS**
N	** **	**LD**	3	5			4	2	** **
	** **	**OS**	1			5			** **
	**S**	**AI**			1	6			**S**
	** **	**LD**			1	5			** **
			**N**	** **	**NS**	** **	**S**	** **	

**Fig 3:**
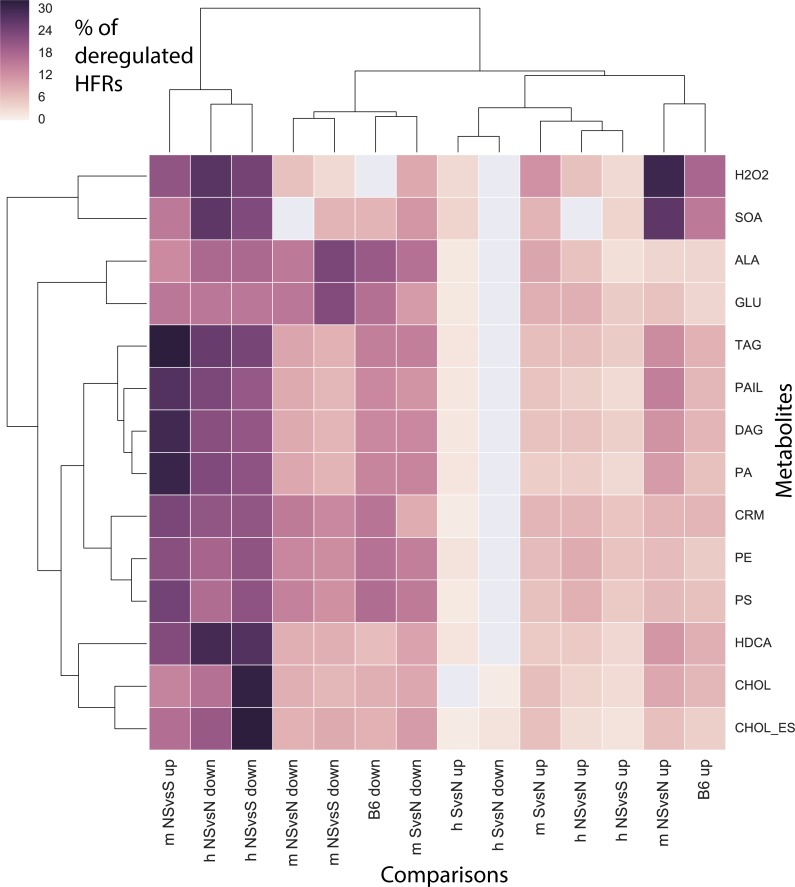
Quantification of up- and downregulated HFRs. Symbols ‘h’ and ‘m’ in comparison’s labels represent human and mouse, respectively, and ‘up’ and ‘down’ indicate up- and downregulation, respectively. See the legend of the Table 3 for the association of mouse strains to the symbols N, NS, and S. “B6 up” and “B6 down” represent the up- and downregulated HFRs for the B6 mouse strain comparing the DDC *vs* control diet. We quantified the percentage of up- and downregulated HFR using a colored heat map.

We further analyzed the deregulation in reaction hubs for each MT to identify the most perturbed MTs in NS and S and the differences between NS and S. In humans, the percentage of downregulated HFRs was higher than upregulated ones for the synthesis of all metabolites in the NS *vs* N and NS *vs* S states, while the percentage of upregulated HFRs was higher than downregulated ones for the S *vs* N state (Fig 3). There was a higher percentage of downregulated HFRs for the cholesterol (CHOL) and cholesteryl ester (CHOL_ES) synthesis networks in NS *vs* S and for the superoxide anion (SOA) and H_2_O_2_ synthesis networks in NS *vs* S (Fig 3). This means that downregulated HFRs have a higher impact on NASH phenotypes.

Since the mouse strains AJ and PWD show phenotypes NS and S, respectively, and the mouse strain B6 shows low S and NS phenotypes after feeding with the DDC diet (Materials and Methods), we analyzed B6 separately. For mice, in NS *vs* N (the comparison associated to AJ) and B6 DDC *vs* N comparisons, we found that the percentage of downregulated HFRs in the SOA and H_2_O_2_ synthesis networks was higher than for upregulated ones (Fig 3). This indicates synthesis networks of oxidative stress were found to be perturbed for AJ and B6 mice. Additionally, for the NS *vs* S in mice, an elevated percentage of upregulated HFRs was identified for the synthesis of the triacylglycerol (TAG), phosphatidylinositol (PAIL), diacylglycerol (DAG), and phosphatidic acid (PA) metabolites; a high percentage of downregulated HFRs was found for the alanine (ALA) and glutamine (GLU) synthesis networks (Fig 3). This is consistent with the observation of lipid droplet perturbation in NASH and steatosis [26], and it further suggests that the synthesis of TAG, PAIL, DAG, and PA are important for lipid droplet formation.

For the synthesis of ROS, such as H_2_O_2_, the human NS *vs* N case presented with a high percentage of downregulated HFRs, while the same case in mice presented with a high percentage of upregulated HFRs (Fig 3). The reactions of the PPP associated with genes TKT1, TKT2, GND, and TALA were identified as downregulated HFRs in the NS *vs* N human. However, for the NS *vs* N mouse and B6 DDC *vs* N these reactions were identified as upregulated. PPP is known as a source of nicotinamide adenine dinucleotide phosphate (NADPH) that prevents oxidative stress. Thus, the different patterns of regulation observed for these reactions can unbalance both NADPH concentration and H_2_O_2_ synthesis. Interestingly it has been reported that the unbalance in NADPH production through PPP by either an under or over-production of NADPH may induce oxidative stress [27, 28]. Interestingly, Mardinoglu and colleagues [29] found an alteration in the hepatic expression levels of associated enzymes of the *de novo* glutathione synthesis and reported that redox imbalance is associated to metabolic dysfunction and the development of NAFLD.

The acetyl CoA C acetyltransferase (ACACT1rm) and phosphoglycerate mutase reactions of the ceramide synthesis networks were identified as downregulated HFRs for the NS *vs* S and NS *vs* N in humans along with the NS *vs* N in mice. Thus, perturbation in the genes associated with these reactions can affect the ceramide level in NASH in both mice and humans. In line with this finding, a study suggested that deregulated ceramide production promotes liver injury and the development of NASH through disruption of endoplasmic calcium homeostasis, as well as through the inhibition of autophagy [30]. Furthermore, another study suggests that the overexpression of acid ceramidase decreases hepatic ceramide levels which is a major contributor of metabolic disease, such as obesity and hepatic steatosis [31].

#### Enriched MTs in human and mouse based on deregulated genes

We want to identify deregulation at network level based on an enrichment analysis with deregulated enzymes or genes. A Minimal network (MiN) of a metabolic task (MT) contains metabolites, active reactions and their associated enzymes in order to fulfill the MT. To identify deregulated MTs and their associated MiNs (see Materials and Methods section for definitions) in steatosis and NASH, we performed Minimal Network Enrichment Analysis (MiNEA) (see Materials and Methods section) using mouse and human expression data. We identified deregulated genes (S1 Text; S1–S3 Tables), and for each set of deregulated genes, we estimated the *p* value based on hypergeometic test.

Applying MiNEA to the NS *vs* N human case, we identified deregulated MiNs which corresponded to the synthesis of H_2_O_2_, PA, and TAG (Table 3; S4 & S5 Tables). We computed the percentage of significantly enriched MiNs from all generated alternatives of each MT and scored the deregulated MiNs using the **A**lternative **Mi**nimal **N**etwork **F**requency (AMiNF), which represents the percent of MiNs with enrichment in deregulated genes. For the NS *vs* N case in humans, H_2_O_2_ synthesis had the highest AMiNF (AMiNF = 0.143) compared to all other deregulated MTs (Table 3), suggesting that the deregulation of H_2_O_2_ metabolism and oxidative stress contributes to NASH. Indeed, a study has shown that a marked alteration of H_2_O_2_ concentration can lead to different types of oxidative stress [27].

**Table 3:** The significantly deregulated MTs across all comparisons based on deregulated genes. The numbers in the table indicate Alternative Minimal Network Frequency (AMiNF). The mouse strains associated with N, NS, and S are described in the legend of Table 2. For the calculation of AMiNF *p* < 0.05 was considered statistically significant.

		M	O	U	S	E
		N	NS	S	MT	
	N					N
	** **			1	ALA	** **
H	** **			1	GLU	** **
U	** **			1	CRM	** **
M	**NS**			0.833	PS	**NS**
A	** **			0.714	PE	** **
N	** **	0.143			H_2_O_2_	** **
	** **	0.016			TAG	** **
	** **	0.004			PA	** **
	**S**		0.7		CHOL	**S**
		**N**	**NS**	**S**		

When we applied MiNEA to NS *vs* S in humans, we found the most deregulated MiNs to be associated with CHOL synthesis (AMiNF = 0.7). Interestingly, TAG synthesis was also deregulated in NS *vs* N, suggesting that lipid droplet formation can be perturbed in the NASH state but through different components.

In mice, we did not observe marked deregulation in NS *vs* N and S *vs* N, but we observed significant deregulation in NS *vs* S for the synthesis of ALA, GLU, ceramide (CRM), PS, and phosphatidylethanolamine (PE) (Table 3), suggesting that these MTs are subject to different perturbations between the NASH and steatosis states.

In humans, MiNEA identified deregulation in the CHOL and TAG synthesis network, which are lipid droplet constituents. Similar deregulations were observed for the lipid droplet constituents in mice through the PS and PE synthesis networks. Thus, because MiNEA was applied with information on deregulated genes, we find that in both humans and mice, lipid droplet formation is perturbed but through different lipid constituents. Interestingly, a study [32] using flux-based analysis of metabolic network identified significantly increased activity of the *de novo* lipogenesis in the patients with high amount of liver fat, indicating an alteration in lipid droplet formation.

#### Enriched MTs in humans and mice based on up- and downregulated reactions

To identify whether a specific up- or downregulated MiN was associated with the NAFLD phenotypes, we performed MiNEA while enriching for up- and downregulated reactions separately (S1 Text).

In humans, we identified upregulated MiNs for the synthesis of PS and PE in N *vs* S (Table 4; S6 & S7 Tables). For the NS *vs* N and NS *vs* S cases, we identified downregulated MiNs associated with the synthesis of CHOL_ES, CHOL, ALA, and GLU (Table 5; S8 & S9 Tables), but none were found to be upregulated. This implies that in NASH the synthesis of cholesterol and the glucogenic amino acids alanine and glutamine are downregulated. Interestingly, in the NS *vs* N case, only 3% of the cholesterol synthesis alternatives were significantly downregulated (Table 5). Although it is a relatively small frequency, it can provide important leads, such as deregulation in metabolic tasks and hypotheses that would have been missed by the commonly used pathway enrichment methods, such as [3, 5]. In general, these studies suggested that MiNEA can identify deregulated alternative MiNs under different conditions. For example, previous pathway analysis methods [3, 5] lack an enumeration of alternatives that would have failed to identify cholesterol synthesis as downregulated in NS *vs* N (Table 5).

**Table 4:** The significantly deregulated MTs across all comparisons based on upregulated reactions. For a detailed understanding, see the legend of Table 2. Numbers of the table represent AMiNF and for the calculation of AMiNF *p* value < 0.005 was used.

		M	O	U	S	E
		N	NS	S	MT	
H	**N**			0.029	GLU	**N**
U	** **			1	PA	** **
M	** **			1	TAG	** **
A	**NS**			1	DAG	**NS**
N	** **			0.979	PAIL	** **
	** **			0.167	PS	** **
	**S**	0.429			PS	**S**
	** **	0.167			PE	** **
		**N**	**NS**	**S**		

**Table 5:** The significantly deregulated MTs across all comparisons based on downregulated reactions. For detail understanding see the legend of Table 4. For the AMiNF calculation *p* < 0.005 was considered statistically significant.

	M	O	U	S	E	
		N	NS	S	MT	
			1		ALA	
			1		CRM	
	N		0.857		PE	N
H			0.833		PS	
U			0.62		CHOL_ES	
M			0.588		GLU	
A		0.034			CHOL_ES	
N				1	ALA	
	NS			1	GLU	NS
				1	CRM	
				0.5	PS	
				0.429	PE	
			1		CHOL_ES	
	S		1		CHOL	S
			0.128		ALA	
			0.004		GLU	
		**N**	**NS**	**S**		

In mice, the MiNs associated with the synthesis of ALA, CRM, PE, PS, CHOL_ES, and GLU were identified as downregulated in NS *vs* N (Table 5). Interestingly, in S *vs* N, only the GLU synthesis network was upregulated (Table 4). For the NS *vs* S case, we found major deregulations. Specifically, we found five upregulated networks (PA, TAG, DAG, PAIL, and PS) and five downregulated (ALA, GLU, CRM, PS, and PE) synthesis networks. Within the alternative MiNs that are used for the synthesis of PS, we found some networks that were upregulated and some that were downregulated. Since there was a higher frequency of downregulated MiNs (AMiNF = 0.5) compared to upregulated ones (AMiNF = 0.167), we could hypothesize that the downregulation of PS synthesis is an important molecular mechanism specific to NASH. Here, pathways enrichment methods [3, 5] lack an enumeration of alternatives that would have failed to give a higher confidence to downregulation of PS synthesis.

The cholesterol, glutamine, and alanine syntheses were found to be similarly deregulated in humans and mice for the NS *vs* N case. In contrast, the PS synthesis was differently deregulated between the two species (Tables 4 and 5). Despite this opposite response in the regulation of the PS synthesis between humans and mice, it appears that the level of PS is perturbed in NASH. Overall, this observation suggests that different regulations in gene expression can translate to different effects on the production of lipid droplet synthesis. Interestingly, Maldonado and colleagues [33] explored the role of physiological adaptation to lipid overload and found that increased fatty acid levels mimicked lipid loading in vitro and drove steatotic response critically. Additionally, a further study suggests high levels of free fatty acids and other lipid metabolites activate mitochondrial dysfunction and stress mechanisms associated to endoplasmic reticulum [34].

### Integration of metabolite concentration data into a thermodynamically feasible metabolic model

The integration of metabolomics can change the size and structure of the MiNs for MTs by eliminating thermodynamically infeasible reaction directionalities. In mice, there are available metabolic profiles for the N, NS, and B6 cases [1]. We integrated these profiles into the iMM1415 [12, 14] and found that, in this case, there are not marked changes in the MiNs and the result in the HFRs, which means we did not find a marked impact on the synthesis networks of NAFLD phenotypes. A further sensitivity analysis can guide future metabolomics studies to target metabolites that have a marked thermodynamic impact on a metabolic network [35]. Thus, the information content of such metabolite sets can be influential and more relevant to the phenotypes under study.

### Other applications and modification of MiNEA

We identified all possible minimal networks of given MTs separately and enforced at least 80% of the maximum synthesis rate. However, the "normal physiology", such as activation of normal liver functions can employ a weighted combination of the networks derived by the alternatives in the set of MTs that are characteristic of the "normal physiology". The exact "weighted combination" of MTs is rather uncertain for all MTs simultaneously, as it could create more artifacts. Therefore, we suggest generating minimal networks of given MTs separately. For a given physiology if the weights of MTs are known, then MiNEA can be modified and extended as a path forward for capturing the physiology.

Gene protein reaction (GPR) rules should be able to resolve the regulation of reaction’s rates, but only if the GPR is well curated and validated with experimental data during the reconstruction process. Based on GPR rules, if a reaction is associated with a mixture of upregulated and downregulated genes, we did not consider the reaction as regulated. Here, we chose a rather conservative threshold to resolve these uncertainties, and provide an *upper bound* with reduced noise due to uncertainty. However, the MiNEA is flexible and can include such improved GPR associations.

We should acknowledge that gene regulation based on mRNA levels is not sufficient to determine reaction rates (fluxes) because post-translational modifications, protein translation alone, and kinetics of the enzymes are really important in determining the mapping between mRNA levels and reaction rates. Depending on the availability of proteomics (and phosphoproteomics) data, and even kinetics (such V_max), they can be integrated within the MiNEA method.

We applied MiNEA for the study of deregulated metabolic processes rather signaling and regulatory processes because metabolism is better characterized, as is shown with the increased availability of GEMS for many organisms [36]. Despite the challenges in reconstructing constraint-based signaling networks, recently such reconstructions have started to become available. Signaling networks for the toll-like receptor (TLR) and epidermal growth factor receptor (EGFR) are now available, and MiNEA can be extended to include these networks [37, 38], meaning that MiNEA could easily be applied to the study of deregulation in signaling networks.

#### Conclusions

In contrast to graph-based methods (GBM) and pathway enrichment analysis (PEA), the methodology introduced here (MiNEA) expands the notion of pathway into a set of mass balanced subnetworks that can be used to understand the carbon, energy, and redox flows from precursor metabolites to target metabolites and complex metabolic tasks. One of the main advantages of MiNEA compared to PEA is that MiNEA attempts an enumeration of alternative minimal networks for each metabolic task (MT), which helps us understand and study the MT flexibility. Although a large number of alternative enumerations for a complex metabolic network can be time-consuming, once the enumeration is completed, MiNEA applies a statistical analysis that is fast and extracts additional information, such as the deregulation of MTs or the deregulation in reaction hubs. As an example of the utility of MiNEA, we identified deregulation in key metabolic network of the ceramide and hydrogen peroxide synthesis for NASH in both humans and mice. We also identified similar deregulation in NASH for the cholesterol synthesis networks in humans and mice, and we found opposite deregulation for the phosphatidylserine synthesis network between humans and mice. It has been reported that significant differences between mouse and human metabolism exist mainly in genes for cytochrome P450 based metabolic reactions, which are key reactions in lipid and cholesterol metabolism [14]. Therefore, in order to generalize these results to human the next step is to account for the differences in cholesterol metabolism. MiNEA is highly applicable for the study of context- or condition-specific metabolism because using this one can identify synthesis networks for any given target metabolite and further can employ condition-specific transcriptomics, proteomics, and metabolomics data.

## Materials and methods

### Microarray gene expression analysis of human liver samples

The microarray gene expression data pertaining to the three diagnostic groups (normal [N], Steatosis [S], and NASH [NS]) were collected from the ArrayExpress public repository for microarray data under the accession number E-MEXP-3291 [15]. We performed an analysis of the differentially expressed genes (DEGS) and a pairwise comparison between diagnostic groups. To control the false discovery rate at level of 0.05, multiple hypothesis testing was used [39].

### Mice phenotypes after feeding a 3,5-diethoxycarbonyl-1,4-dihydrocollidine (DDC)-supplemented diet

NASH phenotypes can be reproduced in mouse models by treatment of chronic intoxication of with 3,5-diethoxycarbonyl-1,4-dihydrocollidine (DDC)-supplemented diet [1]. Three genetically different mouse strains AJ, B6, and PWD were fed with a DDC-supplemented diet. Under DDC-supplemented diet, the steatosis and NASH phenotypes were the most obvious in the PWD and AJ strains, respectively, while for the B6 strain, low levels of the steatosis and NASH phenotypes were observed [1]. The B6 mouse strain showed less induction of the NASH phenotype than the AJ strain [1].

### Association of mouse strains with human diagnoses groups

The normal (N) feeding group is associated with mice fed the control diet. All mice strains, AJ, B6 and PWD, were fed this diet and are labeled as N≈AJ, N≈PWD or N≈B6 to indicate the control diet and mouse strain. After feeding the DDC-supplemented diet, AJ and PWD showed high NASH phenotypes and high steatosis phenotypes, respectively [1]. Thus, under the DDC-supplemented diet, the symbol for steatosis (S) is associated with the PWD mouse strain and the symbol for NASH (NS) is associated the AJ mouse strain (with DDC-supplemented diet: NS≈AJ, S≈PWD). Thus, NS *vs* N represents the AJ mouse strain treated with the DDC diet *vs* the AJ mouse strain fed the control diet, S *vs* N represents the PWD mouse strain treated with the DDC diet *vs* the PWD mouse strain fed the control diet, and NS *vs* S represents the AJ mouse strain treated with the DDC diet *vs* the PWD mouse strain fed the DDC diet.

### Metabolite concentration and RNA-seq gene expression data from mouse liver samples

Metabolite concentration data and RNA-seq gene expression data from mouse liver samples of the three mouse strains (AJ, B6, and PWD) measured under the control and DDC-supplemented diet conditions were collected from the work of Pandey et al. [1]. The R-package “edgeR” [40] was used to identify the differentially expressed genes (DEGs) using three biological replicates of each mouse strain for both the control and the DDC-treated conditions. Strain-wise identification of the DEGs between the DDC-treated and control states was performed separately for each mouse strain. We used the Benjamini-Hochberg procedure implemented in edgeR to control the false discovery rate to a level of 0.05 [39].

### Formulation of metabolic tasks (MTs) based on steatosis and steatohepatitis (NASH) phenotypes

Steatosis can occur due to the accumulation of lipid droplets in the liver, which can subsequently lead to NASH upon liver inflammation [41]. Lipid droplets are composed of many lipid metabolites [42, 43], which are summarized in Table 6. Reactive oxygen species (ROS), such as the superoxide anion, can also damage hepatic membranes and play an important role in the development of NASH [44]. To study these various phenotypes and their role in the various forms of liver disease, the metabolites associated with their various phenotypes were examined. Table 6 summarizes the key metabolites associated with the major liver disease phenotypes: lipid droplets, liver inflammation, apoptosis, and oxidative stress. We investigate the synthesis networks of these metabolites using MiNEA, where we called the synthesis of a metabolite as a metabolic task (MT).

**Table 6:** Metabolites associated with NAFLD phenotypes. This provides a summary of key metabolites associated with the four phenotypes and their corresponding references. Metabolites are taken from the mouse metabolic model of Sigurdsson and colleagues [14]. Production of each of these metabolites represents a metabolic task (MT).

Metabolites	Phenotype	Reference
**Diacylglycerol, phosphatidylethanolamine, cholesteryl esters, triacylglycerol, hexadecanoate, cholesterol, phosphatidic acid, phosphatidylinositol, phosphatidylserine**	Lipid droplets	[42, 43, 45]
**Ceramide**	Inflammation	[30, 45, 46]
**Alanine, glutamine, ceramide**	Apoptosis	[47]
**Superoxide anion, hydrogen peroxide**	Oxidative stress	[44]

### Minimal network enrichment analysis (MiNEA) algorithm

**Fig 4:**
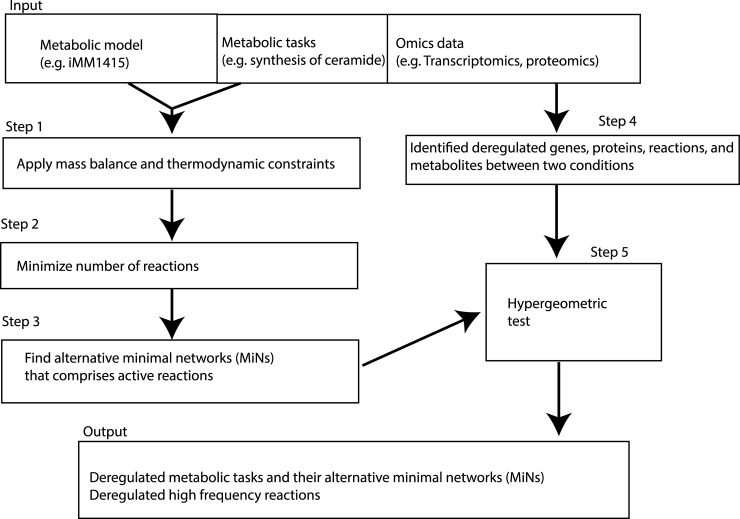
Minimal network enrichment analysis (MiNEA) overview. For each metabolic task (MT), MiNEA computationally enumerates MiNs that comprise active reactions for the MT. The inputs required for the MiNEA analysis are a genome-scale metabolic network (GEM), a given list of metabolic tasks (MTs), and transcriptomics data. Using this, alternative minimal networks (MiNs) are enumerated for MTs using a GEM. (steps 1–3). Transcriptomics data are used to identify differentially regulated genes between two conditions (step 4). To identify deregulated MTs and their associated MiNs, a hypergeometric test is performed with a set of deregulated genes (step 5).

The cell can use various different pathways depending on the current state and conditions of the environment to fulfill its immediate metabolic needs. In order for MiNEA to identify deregulated alternative routes for a given MT between two conditions (Fig 4), the inputs required are a metabolic model, a list of MTs, and gene or protein expression data. In this work, we used a mouse GEM (iMM1415) reconstructed by Sigurdsson and colleagues [14], a list of MTs which are the synthesis of the metabolites shown in Table 6, and gene expression data of mouse and human liver samples [1, 15].

The MiNEA calculations begin by applying thermodynamic constraints to the model as described previously [12] to eliminate thermodynamically infeasible reactions, meaning that reactions from the metabolic network can carry fluxes only if thermodynamics allows [13] (Fig 4; step 1). Then, it enumerates all the thermodynamically feasible minimal-size networks (MiNs) that are active for the given list of provided MTs (Fig 4; step 2), and all of these MiNs are composed of reactions that carry non-zero flux. For this step, the following mixed-integer linear programming (MILP) problem is applied with the objective of minimizing the number of reactions that carry flux or maximizing the number of reactions that cannot carry flux, while enforcing that the network should be able to synthesize the metabolites listed in Table 1:

$$Maximize\sum_{i=1}^{R_{GEM}}z_{rxn,i}$$

S.v=0

$$0\leq v_{i}\leq v_{max,i}*Z_{rxn,i}$$

$$v_{MT}\geq c*V_{MT,max}$$

*R*_*GEM*_ is the number of reactions in a GEM and *z_rxn,i_* is a binary variable associated with reaction *i*. Reversible reactions were split into two forward reactions. *V_MT,max_* is the maximum yield to produce a metabolite associated with a MT. The parameter *c* is generates MiNs that allow for flexibility on the yield from the MT. We chose *c* = 0.8 to allow for a yield of at least 80% of the maximum yield for the associated MT.

All alternative MiNs that are the minimum size (msize) for the synthesis of the metabolites in Table 1 are enumerated as described by Figueiredo and colleagues [48] (Fig 4; step 3). One can enumerate many alternative networks larger than msize, such as msize+1 and msize+2. Each MiN is a subnetwork comprising a set of reactions, metabolites, and reaction-associated genes through gene-protein-reaction (GPR) association. In step 4, the deregulated genes and proteins that differ between the two conditions under study are identified (Fig 4). Finally, a hypergeometric test is performed on the sets of deregulated genes or deregulated reactions to identify the deregulated MiNs (Fig 4; Step 5).

### Minimal network significance based on gene set and reaction set (Step 5)

The significance of the MiN based on the deregulated genes was calculated using the hypergeometric probability density function (P),

$$P(k,N,K,n)=\frac{\left(\begin{array}{c} K\\ k \end{array})(\begin{array}{c} N-K\\ n-k \end{array}\right)}{\left(\begin{array}{c} N\\ n \end{array}\right)},$$ (1)

where *k* and K are the numbers of deregulated genes and the number of total genes in a given MiN, respectively, and n and N are the total number of deregulated genes and the total number of genes of a metabolic network, respectively. In this context, the term “deregulated genes” is used for both up- or downregulated genes.

A reaction can have three states: upregulated, downregulated, or unregulated. The regulation of a reaction was determined based on its associated differentially expressed genes. According to this metric, a reaction was identified as upregulated or downregulated if the corresponding genes are only upregulated or only downregulated, respectively. A reaction that is associated with a mixture of up- and downregulated genes is not characterized as regulated due to the inconsistency of gene expression. Up- and downregulated MiNs, which could contain various combination of up- or downregulated reactions, were identified in Step 4 of MiNEA (Fig 1) based on the total number of up- or downregulated reactions, respectively. As expected, up- and downregulated MiNs comprise markedly high numbers of up- and downregulated reactions, respectively.

The significance of a MiN based on upregulated or downregulated reactions was calculated using multivariate Fisher's hypergeometric distribution. This method has been previously used for the selection of tissue-specific elementary modes using gene expression data [6]. To identify significantly upregulated MiNs in a given set of MiNs, we selected those MiNs that contained an elevated number of upregulated reactions and as few as possible downregulated ones, while for the identification of significantly downregulated MiNs, we selected MiNs with an elevated number of downregulated reactions and as few as possible upregulated ones.

Assume R and T are the total numbers of reactions in a GEM and a MiN, respectively, which can be decomposed as follows:

$$\begin{array}{c} {R=R}_{\mathrm{up}}{+R}_{\mathrm{down}}{+R}_{\mathrm{no}}\\ {T=T}_{\mathrm{up}}{+T}_{\mathrm{down}}{+T}_{\mathrm{no}} \end{array}$$ (2)

*R*_*up*_, *R*_*down*_, and *R*_*no*_ represent the number of upregulated, downregulated, and unregulated reactions in a GEM, respectively. In the context of a MiN, T is the total number of reactions, and *T*_*up*_, *T*_*down*_, and *T*_*no*_ are the number of upregulated, downregulated, and unregulated reactions, respectively.

To consider a MiN as upregulated, we need to ensure that the pair (*T*_*up*_, *T*_*down*_) in the MiN of *T* reactions cannot arise by chance in the context of the whole network. To obtain a better upregulation by chance, the *p* value was computed using Eq (3). Note that an equal or better outcome than the pair (T_up_, T_down_) satisfies two conditions: (i) the number of downregulated reactions is smaller than or equal to T_down_, and (ii) the number of upregulated reactions is greater than or equal to T_up_, whereas the number of reactions in the MiN remains unchanged.

$$pvalue=\begin{array}{l} \sum_{{i=T}_{\mathrm{up}}}^{\min\left(R_{\mathrm{up}},T\right)}\sum_{j=0}^{S_{\mathrm{down}}}\frac{\left(\begin{array}{c} R_{\mathrm{up}}\\ i \end{array}\right)\left(\begin{array}{c} R_{\mathrm{down}}\\ j \end{array}\right)\left(\begin{array}{c} R_{\mathrm{no}}\\ S-i-j \end{array}\right)}{\left(\begin{array}{c} R\\ T \end{array}\right)}\\ \;i+j\leq T \end{array}$$ (3)

We can compute the *p* value for downregulated reactions the same way as for the upregulated reaction simply by changing ***up*** with ***down*** and ***down*** with **up** in the above equation.

### Deregulated percentage (DRP)

We calculated the deregulated percentage (DRP) for each MiN, which indicates the percentage of up- or downregulated genes in a MiN. For example, if a given MiN comprises 20 genes, and 5 genes of the MiN are deregulated, the then DRP is equal to 0.4. Since the active reactions in a MiN were classified as either up- or downregulated, we calculated both upregulated percentage (UPR) and downregulated percentage (DnRP) for each MiN.

### Alternative minimal network frequency (AMiNF)

To extend the degree of confidence of the results from MiNEA, we introduced the alternative minimal network frequency (AMiNF) metric that determines the significantly deregulated percentage of MiNs for a given MT. If all the alternative MiNs for a MT are significantly deregulated, AMiNF takes the value 1. The AMiNF is 0 when none of the MiNs for a MT are significantly deregulated.

### MiNEA implementation

MiNEA was implemented using Matlab r2016a (The Mathworks, Natick, MA, USA), and MILP problems were solved using CPLEX solver (ILOG, Sunnyvale, CA, USA). The MILP gaps for all problems were converged to less than 0.05% in less than 2,400 s. We also plan to make the MiNEA algorithm available as a tool for distribution to the community.

## Supporting information

S1 Table Differentially expressed genes for human and mouse expression data. (XLSX)

S2 Table Upregulated reactions for human and mouse data. (XLSX)

S3 Table Downregulated reactions for human and mouse data. (XLSX)

S4 Table Minimal network enrichment analysis based on differentially expressed genes. Description of the table heading is illustrated in ‘Symbol’ sheet of the excel file. Please see the Symbol sheet for the detail description for the tables: S5-S9.
(XLSX)

S5 Table Minimal network enrichment analysis based on upregulated reactions. (XLSX)

S6 Table Minimal network enrichment analysis based on downregulated reactions. (XLSX)

S7 Table Marked deregulated minimal networks selected based on S4 Table. (XLSX)

S8 Table Marked deregulated minimal networks selected based on S5 Table. (XLSX)

S9 Table Marked deregulated minimal networks selected based on S6 Table. (XLSX)

S1 Fig Venn diagram of differentially expressed genes of human and mouse liver samples. Upper and lower panels represent human and mouse, respectively.
(EPS)

S2 Fig Venn diagram of up- and downregulated reactions of the iMM1415 in human and mouse liver samples. The reaction regulation metric was used to identify up- and downregulated reactions.
(EPS)

S1 Text In this text a detailed description is defined for identifying deregulated genes and deregulated reactions based on human and mouse expression data. (DOCX)
