# Supplementary material for: Investigating the deregulation of metabolic tasks via Minimum Network Enrichment Analysis (MiNEA) as applied to nonalcoholic fatty liver disease using mouse and human omics data
Source: PLoS Comput Biol. 2019 Apr 19;15(4):e1006760. doi: 10.1371/journal.pcbi.1006760 (PMC6493771; doi:10.1371/journal.pcbi.1006760)
Supplement: S1 Text — (DOCX) [file pcbi.1006760.s012.docx]

## **Supporting Information**

**Comparison of human and mouse genome-scale metabolic models**

Mouse genome-scale metabolic model, iMM1415 [1] was reconstructed based on a human genome-scale metabolic model (Recon 1, [2]). Sigurdsson and collogues found that the mammalian organism with the highest number of genes homologous to Recon 1 genes was the mouse (*Mus musculus*) (1,415 genes, 97%). We compared iMM1415 and Recon1 and found that the iMM1415 shares 98% of reactions with Recon1 and in the reaming 2 % reactions more than 1.5 % reactions were associated to the extracellular transport mechanism. This suggests that Recon1 and iMM1415 have very similar metabolism.

## **Identification and analysis of deregulated genes and reactions in human and mouse liver samples**

To understand the nonalcoholic fatty liver disease (NAFLD) and the difference of the disease in mouse and human we collected human expression data form the three diagnosis groups: normal (N), steatosis (S), and nonalcoholic steatohepatitis (NS), and mouse expression data form the control and DDC-supplement diet conditions for the three mouse strains: AJ, B6 and PWD (*Materials and Methods*). These data were referred as *human expression data* and *mouse expression data* throughout this section.

## **Differential expressed genes in human liver samples**

To understand the NAFLD physiology we analyzed the differentially expressed genes (DEGs) in the *human expression data*. Out of the 1415 metabolic genes in iMM1415 [1], we identified 29, 484 and 363 DEGs in S *vs* N, NS *vs* S, and NS *vs* N, respectively (Fig. S1 upper panel and Table S1). Only 29 DEGs between S *vs* N may explain that the metabolic state was very similar between normal and steatosis. The total number of DEGs between NS *vs* N and NS *vs* S were much higher than S *vs* N, and thus suggest a more pronounced alteration of the metabolic state of nonalcoholic steatohepatitis compared to steatosis and normal. Furthermore, the number of downregulated genes is greater than the number of upregulated genes in exclusively NS *vs* N and NS *vs* S (Fig. S1), suggesting that, in human NASH, the perturbation leading to the metabolic state that characterizes it, is reached by downregulated genes.

## **Differential expressed genes in mouse liver samples**

We analyzed the *mouse expression data* for DEGs as described in the *material and methods* section. Out of the 1415 metabolic genes in iMM1415, the total number of DEGs between control and DDC-supplemented diet was similar across all strains with 247, 248 and 221 for AJ, B6 and PWD, respectively (Fig. S2 lower panel). Here, AJ and PWD are associated with NS *vs* N and S *vs* N, respectively (see materials and methods). Many up- and down-regulated genes were strain-specific. The number of up- and down-regulated strain-specific gene pairs for AJ, B6, and PWD were (48, 20), (33, 29), and (39, 62), respectively (Fig. S1 lower panel and Table S1). The number of up-regulated genes was greater than the number of down-regulated genes for the AJ strain, while for the PWD strain the opposite was true. Numbers of up- and down-regulated genes were very similar for the B6 strain. Interestingly, the observed differences qualitatively correlate with the strains’ phenotypes: steatohepatitis phenotypes were observed high, low, and unspecific for the AJ, B6, and PWD mouse strains, respectively [3].

We identified deregulated genes form the AJ under the DDC-supplemented diet *vs* PWD under DDC-supplemented diet and this is associated with NS *vs* S (see materials and methods). For this comparison we identified 191 and 100 up- and down-regulated genes, respectively (Table S1).

## **Up- and downregulated reactions in human and mouse**

A reaction is marked as down regulated if genes associated to the reaction is down-regulated and if genes associated to the reaction is upregulated then the reaction is called as upregulated. If a reaction is associated to genes with mix-regulation (up- and down-regulation) then the reaction is not marked with up- or down-regulated. Regulations of reactions are computed for the *human expression data* and *mouse expression data* (Fig. S2).

Only for NS *vs* S, the number of down-regulated reactions was higher than the number of up-regulated reactions (Fig. S2 upper panel), and a similar trend was observed for the number of up- and down-regulated genes (Fig. S1 upper panel and Tables S2-S3). The number of reactions up- and down-regulated was similar for NS *vs* N, but the number of down-regulated genes was higher than the number of upregulated genes (Fig. S1 and S2 upper panels). For S *vs* N, the number of down-regulated reactions was higher than the number of up-regulated reactions; however, the numbers of up- and down-regulated genes for these conditions were similar (Fig. S1 and S2 upper panels). This observation indicates that the reaction regulation (RR) is not always in agreement with the gene regulation, which results from the dependency on the gene association set rather than a single gene.

For the AJ and B6 mouse strains, we found that the number of upregulated reactions was higher than the number of downregulated reactions, whereas for the PWD mouse strain the inverse was observed (Fig. S2 lower panel). For AJ and PWD, the gene regulation followed the same trend for reaction regulation, whereas for B6 the numbers of up- and down-regulated genes and reactions remained comparable (Fig. S1).

To represents as the human NS *vs* S we compared mice that have shown high nonalcoholic steatohepatitis (NS) phenotypes (AJ mice fed with the DDC-supplemented diet) to mice that have shown high steatosis (S) phenotypes (PWD mice fed with DDC supplemented diet). For the NS *vs* S in mice (see materials and methods; AJ DDC vs PWD DDC) we identified 459 and 191 up- and down-regulated reactions, respectively (Table S 2-S3).

**Supplementary Figures**

Figure S1. Venn diagram of differentially expressed genes of human and mouse liver samples. Upper and lower panels represent human and mouse, respectively.

Figure S2. Venn diagram of up- and downregulated reactions of the iMM1415 in human and mouse liver samples. The reaction regulation metric was used to identify up- and downregulated reactions.

1. Sigurdsson MI, Jamshidi N, Steingrimsson E, Thiele I, Palsson BØ. A detailed genome-wide reconstruction of mouse metabolism based on human Recon 1. BMC systems biology. 2010;4:140. doi: 10.1186/1752-0509-4-140.

2. Duarte NC, Becker SA, Jamshidi N, Thiele I, Mo ML, Vo TD, et al. Global reconstruction of the human metabolic network based on genomic and bibliomic data. Proceedings of the National Academy of Sciences of the United States of America. 2007;104(6):1777-82. doi: 10.1073/pnas.0610772104. PubMed PMID: WOS:000244127900011.

3. Pandey V, Sultan M, Kashofer K, Ralser M, Amstislavskiy V, Starmann J, et al. Comparative Analysis and Modeling of the Severity of Steatohepatitis in DDC-Treated Mouse Strains. PLoS ONE. 2014;9:e111006. doi: 10.1371/journal.pone.0111006.
